# The neuroscience of positive memory deficits in depression

**DOI:** 10.3389/fpsyg.2015.01295

**Published:** 2015-09-07

**Authors:** Daniel G. Dillon

**Affiliations:** Motivated Learning and Memory Laboratory, Center for Depression, Anxiety and Stress Research, McLean Hospital, Harvard Medical School, Belmont, MA, USA

**Keywords:** depression, reward, memory, anhedonia, hippocampus

## Abstract

Adults with unipolar depression typically show poor episodic memory for positive material, but the neuroscientific mechanisms responsible for this deficit have not been characterized. I suggest a simple hypothesis: weak memory for positive material in depression reflects disrupted communication between the mesolimbic dopamine pathway and medial temporal lobe (MTL) memory systems during encoding. This proposal draws on basic research showing that dopamine release in the hippocampus is critical for the transition from early- to late-phase long-term potentiation (LTP) that marks the conversion of labile, short-term memories into stable, long-term memories. Neuroimaging and pharmacological data from healthy humans paint a similar picture: activation of the mesolimbic reward circuit enhances encoding and boosts retention. Unipolar depression is characterized by anhedonia–loss of pleasure–and reward circuit dysfunction, which is believed to reflect negative effects of stress on the mesolimbic dopamine pathway. Thus, I propose that the MTL is deprived of strengthening reward signals in depressed adults and memory for positive events suffers accordingly. Although other mechanisms are important, this hypothesis holds promise as an explanation for positive memory deficits in depression.

## Introduction

Unipolar depression impairs episodic memory (Burt et al., 1995; Zakzanis et al., 1998), and the dominant hypothesis is that stress-induced changes in the hippocampus are responsible (Sapolsky, 1996, 2000; Sheline et al., 1999; MacQueen et al., 2003; MacQueen and Frodl, 2011; Huang et al., 2013; Travis et al., 2014). The idea is straightforward: stress is a potent risk factor for depression (Kendler et al., 1999; Monroe and Harkness, 2005), and the dense concentration of glucocorticoid receptors in the human hippocampus (Wang et al., 2013) makes it a prime candidate for stress-induced neurotoxicity^1^. Indeed, the hippocampus is smaller in adults with recurrent depression (MacQueen and Frodl, 2011), and post-mortem exams reveal shrunken hippocampal neurons and glial cells in depressed individuals (Stockmeier et al., 2004). Because the hippocampus is the seat of episodic memory (Squire, 1992), a causal chain running from stress to depression to hippocampal volume reductions provides an appealing account of memory deficits in depression.

However, there are reasons to think the hippocampal stress hypothesis would benefit from supplementation. First, although adults with recurrent depression typically show memory deficits and hippocampal volume reductions, strong evidence for a direct relationship between these two phenomena is lacking (MacQueen et al., 2003; Travis et al., 2014). Second, research on emotional memory suggests an important role for neural mechanisms implicated in positive emotional responses. Excessive sadness is one of two cardinal symptoms of major depressive disorder (MDD; American Psychiatric Association, 2013), and since there is a tendency for depressed adults to preferentially attend to and ruminate on negative information (Beck et al., 1979; Nolen-Hoeksema, 1991), one might expect better memory for negative material in depressed adults, as information consistent with one’s mood is preferentially encoded (Bower, 1981, 1987). Indeed, this effect is often found (e.g., Gotlib et al., 2004; Hamilton and Gotlib, 2008; Matt et al., 1992)—but positive memory deficits are also robust. Healthy adults often show better memory for positive versus negative or neutral material, but this advantage is reduced in depression (e.g., Gotlib et al., 2004; Hamilton and Gotlib, 2008), and a seminal meta-analysis found that this group difference is more reliable than enhanced memory for negative material in depression (Burt et al., 1995). Why is memory for positive material impaired in depressed adults? I propose that it reflects anhedonia—the second cardinal symptom of MDD—and its association with dysfunction in mesolimbic dopamine circuits that respond to reward (Schultz, 1998).

In this article I review key studies from cellular, behavioral, and human neuroscience that underscore the critical role of dopamine transmission in the persistence of episodic memories (for more extensive reviews, see Frey and Morris, 1998a; Lisman and Grace, 2005; Shohamy and Adcock, 2010; Lisman et al., 2011). When considered alongside growing evidence of reward system dysfunction in depression (Eshel and Roiser, 2010; Treadway and Zald, 2011; Dillon et al., 2014b), these data invite the following inference: memory for positive material is impaired in depressed adults because, on average, mesolimbic dopamine circuits do not mount an adequate response to reward in such individuals, compromising interactions between reward and memory systems that ensure memory retention. As stress is the most likely cause of weak dopaminergic reward responses in depression (Dillon et al., 2014b; Pizzagalli, 2014), this hypothesis extends the existing literature: stress cannot only induce hippocampal volume reductions, it can also perturb reward circuits and preferentially disrupt the formation of positive memories.

## Synaptic Tagging and Capture

The synaptic tagging and capture (STC) hypothesis forms the foundation of this proposal (Frey and Morris, 1997, 1998a,b; Frey and Frey, 2008). STC solves a routing problem confronted by the brain’s cellular learning and memory mechanism, long-term potentiation (LTP; Bliss and Lømo, 1973). To appreciate the nature of the problem, consider a population of hippocampal neurons communicating across a synapse. If an experimenter applies weak electrical stimulation to the pre-synaptic neurons and measures the post-synaptic response, she will observe an increase in activation (specifically, excitatory field potentials) that will decay back to baseline within about 3 to 6 h; this transient response is called early-LTP (Frey and Morris, 1997). But if she applies strong stimulation, she will record increased post-synaptic activation that can be maintained for days, weeks, or months (Abraham, 2003); this sustained response is called late-LTP. Late-LTP reflects the operation of molecular processes that structurally remodel the connection between pre- and post-synaptic neurons, transforming a country path into an eight-lane highway and making information trafficking easier (Baudry et al., 2011). The routing problem arises because most of the plasticity-related proteins (PRPs) needed for remodeling are synthesized in the body of the neuron, but LTP is synapse-specific. There are thousands of synapses per neuron (Pakkenberg et al., 2003), all of them far downstream from the cell body, so getting PRPs to the right synapses is a significant challenge. In other words, if late-LTP amounts to building a bridge between neurons, then the concrete is mixed at a rural plant and must be delivered to a construction site in a busy neighborhood downtown—how does the delivery driver find his way?

**FIGURE 1:**
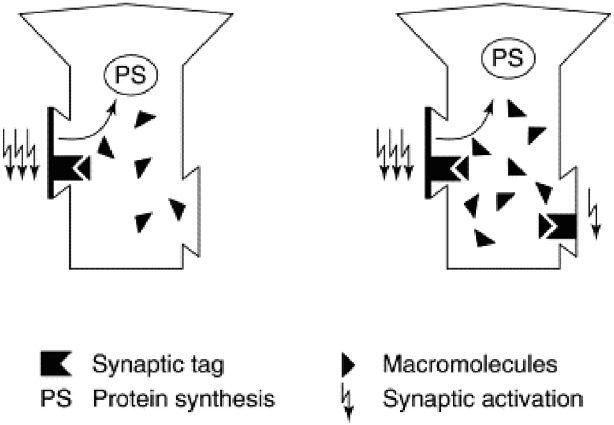
**Synaptic tagging and capture.** Strong activation of a synapse (left panel, three lightning bolts) results in the formation of a synaptic tag and causes protein synthesis in the cell body. Plasticity related proteins (“macromolecules”) then travel from the cell body and can be captured by tag-bearing synapses; once captured, they mediate the transition from early-LTP to late-LTP. Weak synaptic activation (right panel, single lightning bolt) is sufficient to set a synaptic tag but will not elicit protein synthesis. However, a tag set by weak activation can capture plasticity related proteins formed in response to strong activation (right panel, three lightning bolts). Because dopamine release can trigger protein synthesis, synaptic tagging and capture is a mechanism that can explain how the events that elicit dopamine bursts—and events that simply occur in temporal proximity to dopamine bursts—are typically well-encoded and retained in memory. Reprinted from Frey and Morris (1998a) with permission from Elsevier.

Synaptic tagging and capture provides an appealing answer (Figure 1). It turns out that even weak activation is sufficient to set molecular tags that mark a synapse as a candidate for strengthening. If only weak activation occurs, PRPs will not be synthesized and the tags will fade away, consistent with the transient nature of early-LTP (Frey and Morris, 1998a,b; Rogerson et al., 2014). However, if PRPs are synthesized they can begin their trek down the axon without a determined destination, simply stopping at any synapse that displays a tag. When they do so, structural remodeling occurs and a stronger synaptic connection is in place: late-LTP.

This raises another issue. Neurons use an on-demand inventory system and synthesize PRPs as they are needed. In the laboratory, strong electrical stimulation drives PRP synthesis, but what triggers their production in nature?

Dopamine is critical (Frey and Morris, 1998a; Smith et al., 2005; Lisman et al., 2011). Direct evidence for this claim comes from an *in vitro* study that used fluorescence imaging to track the production of new proteins in hippocampal neurons (Smith et al., 2005). This work showed that applying D1/D5 dopamine receptor *agonists* to the hippocampus results in increased protein synthesis, an effect that can be blocked by applying D1/D5 receptor *antagonists*. Furthermore, the newly synthesized proteins included a subunit of the AMPA receptor, which is a key contributor to late-LTP: increased post-synaptic AMPA receptor density is a major part of the “bridge” between neurons (Malinow, 2003). Finally, if dopamine-driven protein synthesis contributes to LTP, then one would expect application of D1/D5 receptor agonists and antagonists to influence the post-synaptic response to pre-synaptic stimulation in opposite directions. Indeed, this was observed: for a given level of stimulation, application of the agonists doubled the response frequency of post-synaptic neurons, but application of the antagonists blocked this effect. This is compelling evidence that hippocampal dopamine release is crucial for the synthesis of proteins that mediate late-LTP.

There is also a wealth of evidence regarding the role of dopamine in the synthesis of PRPs from studies that inferred their presence (or absence) by examining late-LTP. For instance: (1) the concentration of hippocampal dopamine increases following late-LTP induction (Frey et al., 1990); (2) the application of D1/D5 receptor agonists can directly induce late-LTP, skipping early-LTP entirely (Huang and Kandel, 1995); and (3) the oral administration of L-DOPA—a dopamine precursor used to treat Parkinson’s Disease—lowers the stimulation necessary to transition from early to late-LTP in the rodent hippocampus, as does application of a D1/D5 receptor agonist (Kusuki et al., 1997). All of these effects can be blocked by administration of D1/D5 receptor antagonists or protein synthesis inhibitors.

In summary, dopamine release drives PRP synthesis, which enables the transition from early to late-LTP (Frey and Morris, 1998a). Critically, any event capable of driving dopamine release is also strong enough to place tags on the synapses it activates. Since PRPs are sequestered by tag-bearing synapses, this is a mechanism for memory formation: dopamine release stabilizes LTP for the events that caused its release. If dopamine release is disrupted, memory for those events will suffer accordingly. Because many of the events that cause dopamine release also elicit positive emotional responses, disrupting the mesolimbic dopamine circuit should preferentially impair long-term memory for emotionally positive events.

## Dopamine Supports the Retention of Episodic Memory in Non-human Animals

One could accept the findings reviewed above and yet question whether dopamine is relevant to episodic memory. After all, most of the work just described was conducted *in vitro* rather than in behaving animals. Even if dopamine proves important for hippocampal function in rodents, classic accounts of episodic memory hinge on conscious experience (Tulving, 1993). Given the challenges associated with assessing consciousness in lab rats, one could fairly ask: Can we study episodic memory in animals?

The answer is yes. An alternative approach to episodic memory conceptualizes it not in terms of consciousness, but rather as memory for events-in-context: not only knowing that an event happened, but also knowing the spatial and temporal circumstances in which it happened (Clayton and Dickinson, 1998; Allen and Fortin, 2013). In other words, memory for an episode, defined as events taking place in a certain space or time, or in a particular sequence (MacDonald et al., 2013).

An elegant study used a method that met these criteria to show that memory persistence depends on the activation of hippocampal dopamine receptors (Bethus et al., 2010). The study used a sand-filled rectangular “event arena” with six wells in which rats could dig. On each training trial, rats were placed in one of four start boxes and given a food pellet with one of six flavors. The rat was then allowed to enter the arena and search for food. Critically, the “cue” pellet given in the start box determined which well contained additional pellets. The pairing of cues to wells was stable, and after 16 days the rats were running to the correct wells on about 80% of trials. This degree of accuracy is especially impressive because the use of four start boxes located in different places meant that a strategy based on landmarks would necessarily fail. Instead, the animals must have developed a map into which the cue-well associations were embedded.

With training complete, the experimenters tested episodic memory by introducing a novel cue flavor and placing two new wells in the arena, along with the original six. Only one of the two new wells was loaded with pellets. The rats explored the arena until they found the pellets, thus encoding a new cue-well association. Next, an identical trial was used to test memory, following delays of either 30 min or 24 h. Rats showed excellent memory regardless of the delay, running to the new cued well and avoiding both the new uncued well and the original six wells. However, performance after 24 h was at chance following hippocampal lesions administered after training but prior to encoding (Tse et al., 2007), and a similar impairment was observed when NMDA receptor blockers were injected into the hippocampus (Bethus et al., 2010). Because activation of NMDA receptors is essential to LTP (Lynch, 2004), these results indicate that 24-h memory for material learned in the event arena requires hippocampal LTP. In other words, performance in this task displays the hallmarks of episodic memory: rapid encoding of events-in-context, with long-term retention dependent on LTP in the hippocampus.

**FIGURE 2:**
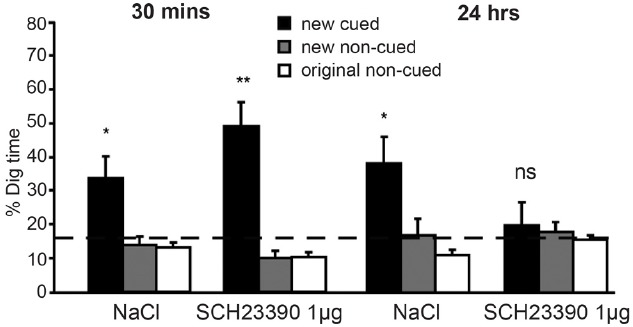
**Dopamine release in the hippocampus is critical for long-term retention of episodic memories in rodents.** In the event arena, injection of the D1/D5 receptor antagonist (SCH23390) into the hippocampus prior to encoding did not affect episodic memory when a retrieval test was given after a 30-min delay. However, a strong negative effect was seen after 24 h. At this time point, animals who had received saline (NaCl) injections continued to perform adequately, but those who had received SCH23390 injections performed at chance levels. Reprinted from Bethus et al. (2010) with permission from the Society for Neuroscience. *p < 0.05, **p < 0.01, ns, means non-significant.

Having established that (episodic) memory for novel cue-well pairs depends on hippocampal LTP, the experimenters next demonstrated a critical role for dopamine in memory persistence (Figure 2). When they injected D1/D5 receptor antagonists into the hippocampus prior to encoding and tested retrieval after a 30-min delay, performance was unaffected. However, delaying the retrieval test by 24 h revealed a profound impairment, with performance no better than chance: given the new cue, rats ran to the uncued well as often as to the cued well (importantly, memory for the original six cue-well pairings was unimpaired). Control experiments showed that this effect did not reflect state-dependent retrieval: if the antagonists were injected into the hippocampus at encoding then 24 h memory was impaired whether or not the antagonists were also injected at retrieval, and injecting the antagonists only at retrieval had no effect. In other words, using a drug to block dopamine release in the hippocampus at encoding impaired long-term memory, and this effect could not be rescued by injecting the drug again just prior to the memory test. These data extend *in vitro* studies reviewed in the prior section to behavior *in vivo*: just as dopamine is more important for late than early LTP, so dopamine release in the hippocampus is more important for long-term versus short-term retention of episodic memories (for additional evidence, see Wang et al., 2010).

One need not inject dopaminergic agents to observe these effects, as well-chosen behavioral interventions yield similar results. For instance, novelty elicits mesolimbic dopamine release in non-human animals (Lisman and Grace, 2005), and exposure to novelty enhances memory persistence in the event arena. In a modified version of the task, rats encoded a new association between a cue flavor and the location of a single well—the only well available—before completing a cued retrieval test in which six wells were available (Wang et al., 2010). This procedure was repeated daily for 6 months, with a different cue-well association learned and tested each day (the experimenters drew an analogy to parking one’s car in different spots within the same lot). When a single pellet served as the reward for finding the correct well during learning, retrieval for cue-well pairings was adequate after a 30-min delay but decayed substantially after 24 h. However, 24-h memory was rescued by a simple manipulation: exposing the rats to a novel environment 30 min after learning.

Synaptic tagging and capture can account for this finding. Encoding the cue-well pairing results in synaptic activation that is sufficient to set a tag but not strong enough to drive PRP synthesis because of the meager reward (1 pellet) delivered for finding the correct well. Consequently, if nothing else happens, late-LTP will not occur and the cue-well memory will fade away within 24 h. However, exposure to a novel context drives dopamine release in the mesolimbic circuit, and this is sufficient to trigger PRP synthesis. The molecular tags set during cue-well learning will not fade in the 30 min post-encoding, so when novelty exposure triggers dopamine release and PRP synthesis, the tags will still be set and can sequester PRPs, leading to late-LTP and robust 24 h memory. This account was strengthened by the fact that the positive effect of novelty on memory retention was blocked if novelty exposure was accompanied by the injection of D1/D5 receptor antagonists or protein synthesis inhibitors; either of these agents can disrupt PRP synthesis and thus deny the synapse a chance at late-LTP.

Note that enhanced memory persistence following a post-encoding manipulation (here, novelty exposure) is consistent with STC: as long as synaptic tags are present when PRPs are made available, the transition from early to late-LTP will occur and a lasting memory will be formed, regardless of whether the PRPs are synthesized before or after the tags are set (Frey and Morris, 1998a). STC also predicts a time window governed by the decay of the tags, and this study found evidence for such a window: novelty exposure 6 h post-encoding did not rescue 24-h memory and injecting protein synthesis inhibitors 6 h post-encoding did not impair 24-h memory. Thus, tag setting and PRP capture are complete within 6 h after encoding (other experiments find evidence for considerably more narrow windows, e.g., 2 h in Moncada and Viola, 2007).

Finally, using larger reward (3 pellets) at encoding had the same effect as novelty exposure: memory accuracy was sustained at the 24-h test, and injection of D1/D5 receptor antagonists into the hippocampus blocked this effect. Remarkably, this blockade of “strong” reward memory could be prevented by novelty exposure. When rats explored a novel environment prior to completing encoding trials in which 3 pellet reward were given, 24-h memory was intact even if encoding occurred under dopamine receptor blockade. This confirms a central prediction of STC: once PRPs are made available—here, by novelty exposure—they can be captured by tags that are set shortly afterward, even if PRP production is blocked during tag setting (see also Moncada and Viola, 2007; Ballarini et al., 2009).

In summary, rodents can form episodic memories, the persistence of which depends on dopamine release into the hippocampus. Furthermore, memory strength can be bolstered by behavioral manipulations that trigger dopamine release, such as the opportunity to explore a new environment or the receipt of 3 pellets rather than one for successful encoding. The next section highlights the fact that these manipulations have similar effects in humans.

## Anticipation of Reward and Novelty Support Episodic Memory in Healthy Humans

A role for dopamine in episodic memory formation is consistent with anatomical studies in non-human animals (Lisman and Grace, 2005). In the rodent, there are direct projections from the ventral tegmental area (VTA) to the hippocampus, along with indirect projections from the hippocampus to the VTA that go through the nucleus accumbens and pallidum. The VTA-to-hippocampus connection permits the dopaminergic modulation of hippocampal LTP that has been discussed thus far. Meanwhile, the hippocampal-to-VTA connection is critical for triggering dopamine bursts in the first instance. The hippocampus holds a representation of the current context and detects deviations from that context. When a novel stimulus or an unexpected reward is detected, the hippocampus registers the deviation and transmits that information to the VTA, leading to burst firing of dopamine neurons.

Do humans have the same pathways? Definitive anatomical studies have not been done, but an investigation of spontaneous functional connectivity in fMRI data is suggestive (Kahn and Shohamy, 2013). In two large samples (*n* = 100 and *n* = 894), analysis of resting state fMRI signals revealed significant correlations among the body of the hippocampus, the nucleus accumbens, and the VTA. Although functional connectivity does not imply anatomical connectivity, the fact that correlated activation among these regions was detectable at rest is encouraging because it implies synchronous patterns of activation in the absence of external stimulation, which suggests stable communication between these regions in humans.

Furthermore, pharmacological work in humans has shown that dopaminergic agents can influence episodic memory. Specifically, enhancing dopamine transmission improves memory in a manner that appears consistent with STC. Chowdhury et al. (2012) administered L-DOPA to healthy older adults prior to an encoding session in which they viewed two categories of images, with one category reliably predicting delivery of monetary reward. Memory for half the items was tested after a 2-h delay, with memory for the remaining items tested after a 6-h delay. The study generated a striking finding—namely, a quadratic relationship between L-DOPA levels and delayed memory for neutral images (i.e., images that did not predict reward delivery), such that a moderate dose of L-DOPA improved 6-h memory for the neutral images relative to small or large doses. No such curve was evident after the shorter delay or for reward-predicting images at either delay. This is intriguing because 6-h memory for neutral images is the condition in which maximal forgetting would be expected, and thus it is also the condition in which heightened levels of dopamine could most easily rescue performance, much in the way that novelty exposure rescued 24-h memory in the 1-pellet encoding condition tested by Wang et al. (2010), as described earlier.

Additional pharmacological studies in humans are needed, as most of the human data pertinent to dopamine and memory come from task-based fMRI research (for review, see Shohamy and Adcock, 2010). Of course, fMRI cannot directly measure dopamine transmission and interpretations should be cautiously made (but for evidence of a relationship between dopamine receptor occupancy and fMRI signal from simultaneous fMRI/PET, see Mandeville et al., 2013). Nonetheless, the parallels with rodent data are striking. The earliest work on this topic demonstrated activation of the hippocampus and midbrain, including the VTA and substantia nigra (SN), in response to novel configurations of familiar images and during the encoding of successfully recalled words (Schott et al., 2004). Robust hippocampal activation was also observed, consistent with the hypothesis that these two regions form a functional unit in humans as well as in rodents.

The interpretation of these data is predicated on the hypothesis that the human mesolimbic dopamine network responds to novelty in much the same way as it responds to reward. A subsequent study from the same team provided compelling evidence for this hypothesis. The dopaminergic midbrain fires strongly to reward-predicting cues and to unexpected reward delivery (Schultz, 1998). To determine whether the human midbrain shows similar functionality with respect to novelty, Wittmann et al. (2007) presented participants with two colored squares that predicted the appearance of novel and familiar images, respectively. The predictions were accurate 75% of the time; on the remaining 25% of trials, unexpected novel or familiar pictures were presented. As hypothesized, fMRI data from the VTA/SN showed a strong response to the novelty-predicting cue and to the unexpected delivery of novel pictures following the familiarity-predicting cue. This is the pattern expected from the reward literature. Meanwhile, the bilateral hippocampus showed a strong response to the cue predicting novel (versus familiar) images, and activation of the VTA/SN and right hippocampus in response to the novelty-predicting cue was correlated across participants. Finally, memory was tested after a 24-h delay, and higher rates of recollection versus familiarity were observed for expected versus unexpected novel pictures. Taken together, the fMRI and behavioral data suggest that coactivation of the VTA/SN and hippocampus during novelty anticipation—prior to image presentation—facilitated encoding success.

**FIGURE 3:**
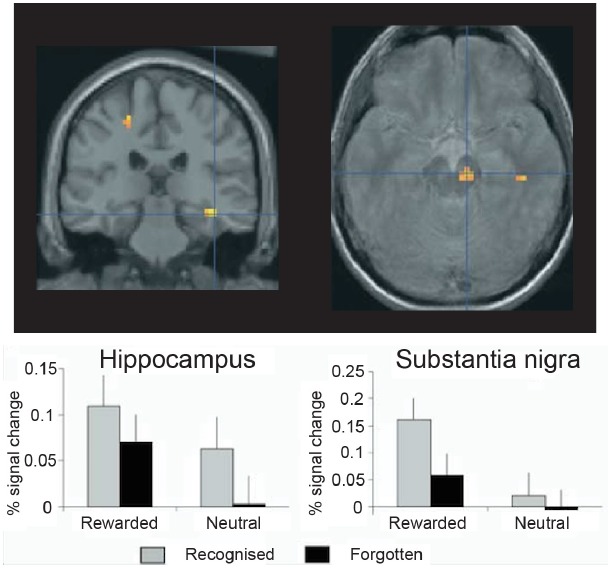
**Encoding activation in the human hippocampus and dopaminergic midbrain predict episodic memory after 3 weeks’ delay.** In the hippocampus, stronger encoding activations was observed for pictures that predicted reward delivery and for pictures that were subsequently recognized, but no interaction was observed. By contrast, in the midbrain [including the substantia nigra and ventral tegmental area (VTA)], a *Reward* × *Memory* interaction was seen: encoding activations was highest for reward predicting pictures that were ultimately remembered. Thus, in healthy controls the midbrain and medial temporal lobe memory regions appear to work together to support episodic memory for images that predict reward delivery. Reprinted from Wittmann et al. (2005) with permission from Elsevier.

Additional fMRI research has found evidence consistent with this interpretation, although it has used monetary reward rather than novel images to drive the VTA/SN (Figure 3). For example, in another study, Wittmann et al. (2005) presented images from two categories, only one of which reliably predicted a chance to win money, and then tested memory for the images at two times: immediately following encoding and again 3 weeks later. The fMRI data showed a strong VTA/SN response to the reward-predicting images, which were better remembered than the non-rewarded images when memory was tested after 3 weeks but not when memory was tested immediately. Moreover, when the experimenters probed brain regions whose encoding activation predicted 3-week memory, they found activation in the VTA/SN and the hippocampus. Furthermore, VTA/SN responses showed a *Reward* × *Memory* interaction: activation was higher for subsequently remembered images that predicted reward delivery relative to both images that (1) did not predict reward and (2) predicted reward but were ultimately forgotten. These data indicate that long-term memory is supported by encoding activation in the hippocampus and the dopaminergic midbrain, and they highlight reward anticipation as a potent means for driving the human VTA/SN (see also Adcock et al., 2006; Wolosin et al., 2012, 2013).

Why does the prospect of earning a reward influence encoding? Murty and Adcock (2014) advance the following argument with respect to the incidental encoding of task-irrelevant but salient events: if you are pursuing a valuable goal and you notice something unusual, it may be worth remembering in case it bears on goal-attainment. To test this hypothesis, they showed participants high ($2.00) and low ($0.10) value cues before repeatedly presenting color versions of trial-unique “target” images. The participants’ task was to press a button when the target image changed from color to grayscale. On a subset of trials, a novel image was inserted into the series of targets, allowing Murty and Adcock to pose this question: Are participants more likely to remember novel images following presentation of the high versus the low-value cue? The authors predicted that this effect would emerge, based on the hypothesis that the hippocampus would signal expectancy violations (random presentation of a novel image in a repeating sequence) especially vigorously when a desired goal was at stake.

A memory test administered 30 min later confirmed expectations: memory was better for novel images presented after the high-value versus the low-value cue. Furthermore, only one brain region showed a stronger response to novel images following high- versus low-value cues—namely, the left hippocampus. Hippocampal activation was predicted by the VTA response to high-value cues, with subsequent analyses indicating that the VTA-to-hippocampal relationship was not direct, but was instead mediated by several cortical regions, including the visual cortex, medial PFC, ventrolateral PFC, and subgenual cingulate. In summary, this work showed that reward anticipation can serve as a context that facilitates incidental encoding, and it also provided a rationale for the existence of this mechanism: the brain is frugal, allocating memory space to unexpected events only if it seems like they might help the organism reap reward more effectively.

Overall, the human literature is consistent with studies in hippocampal slices and rodents. Novel configurations of familiar stimuli, stimuli presented when novelty is expected, and images shown during reward anticipation are all well-retained after a delay, although dissociating the effect of these manipulations on short- versus long-term has not received as much attention as in the rodent literature. Limited data from pharmacological studies indicate that dopamine transmission may drive these effects, and fMRI data are consistent with this hypothesis, as they show that coactivation of the hippocampus and dopaminergic midbrain supports memory in these tasks. Although more research is needed, the existing data indicate a similar role for dopamine vis-à-vis memory persistence in humans and rodents.

## Depression

Based on the evidence reviewed thus far, disruption of the mesolimbic dopamine pathway should have significant, negative consequences for episodic memory in humans, with stronger negative effects on long-term versus short-term memory. Because reward delivery is a potent trigger of dopamine release, memory for positive (i.e., rewarding) events should be preferentially disrupted. The putative relationship between anhedonic depression and dysfunction in mesolimbic dopamine circuitry is now widely-known and has been the subject of numerous reviews (Eshel and Roiser, 2010; Treadway and Zald, 2011; Dillon et al., 2014b; Pizzagalli, 2014), to which the interested reader is directed. The novel question at hand is whether or not this dysfunction has the expected effect on memory for rewarded/positive material. Surprisingly, there is virtually no work on this topic. However, a recent study from our group yielded encouraging results.

**FIGURE 4:**
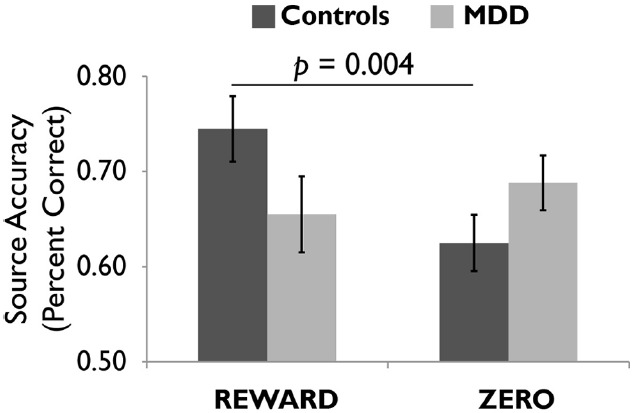
**Healthy controls showed more accurate episodic memory for images followed by reward versus zero tokens, but this effect was not observed in unmediated adults diagnosed with major depressive disorder (MDD).** Reprinted from Dillon et al. (2014a) with permission from Oxford University Press.

**FIGURE 5:**
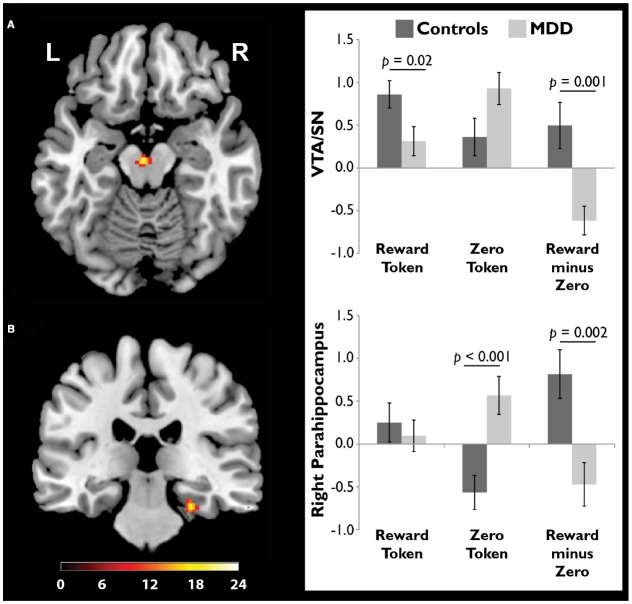
**Compared to adults diagnosed with major depressive disorder (MDD), healthy controls showed a stronger response to reward tokens in the ventral tegmental area/substantia nigra (VTA/SN).** In both the **(A)** VTA/SN and **(B)** right parahippocampus, controls showed stronger activation in response to reward versus zero tokens, while the opposite pattern was observed in the MDD group. In controls but not depressed participants, the VTA/SN “reward minus zero” activation difference score was significantly, positively correlated with the memory advantage for rewarded images (data not shown). Reprinted from Dillon et al. (2014a) with permission from Oxford University Press.

We scanned healthy controls and unmedicated, depressed adults as they viewed drawings followed by reward and zero (non-reward) tokens (Dillon et al., 2014a). A source memory test administered directly after encoding revealed better memory for rewarded versus non-rewarded drawings in the controls, but this effect was absent in the depressed group (Figure 4), consistent with the loss of the positive memory advantage in depression (Burt et al., 1995). We also found a stronger response to the reward versus zero tokens in the VTA/SN and right parahippocampus in the controls, but not the depressed group (Figure 5). Finally, the memory advantage for rewarded versus non-rewarded stimuli was strongly correlated with encoding activation in the VTA/SN in the controls, but no correlation was observed in the depressed adults. Thus, we obtained evidence consistent with the hypothesis that disrupted activation of the VTA/SN and MTL memory regions compromised episodic memory for rewarded material in depressed adults relative to controls.

However, there is much work to be done. Our study—like many studies in the human literature on reward and memory—tested memory directly after encoding. Future studies in depression should test memory after a delay of at least 6 h, as this would give dopamine a chance to exert its effects on the transition from early- to late-LTP. Furthermore, an incidental encoding design—where memory testing is unannounced beforehand—would be preferable (participants in our study knew their memory would be tested prior to encoding). This is because intentional encoding designs like the one we used invite group differences in encoding strategy, which make accurate interpretation challenging. Finally, although it is not possible to directly assess dopamine transmission using fMRI, the reinforcement learning literature has productively used computational modeling to extract a putative dopamine signal from fMRI data (O’Doherty et al., 2007), and there is reason to believe that a computational approach to psychiatric disorders will prove fruitful (Maia and Frank, 2011). In particular, computational modeling may be able to provide increasingly sensitive tests of the proposed role for dopamine abnormalities in human episodic memory failures (for theory on the role of prediction errors in memory, see Henson and Gagnepain, 2010). For example, one could look for a positive relationship between (positive) prediction errors at encoding and accuracy on delayed memory tests in healthy controls, since positive prediction errors are known to elicit burst firing in VTA dopamine neurons (Schultz, 1998). Obtaining such evidence would then allow a test of the hypothesis that this relationship is disrupted in unipolar depression, either because prediction errors are not generated appropriately in depressed adults, or because, once generated, they are not signaled effectively to medial temporal lobe memory regions.

At this point some readers may wonder whether this hypothesis, even if true, is clinically relevant. After all, the cardinal symptoms of major depression are excessive sadness and anhedonia, not memory deficits, and most people probably do not think of depression as a memory disorder. However, research with patients tells a different story. MacQueen et al. (2002) administered a survey to 100 outpatients and found that memory problems were rated as the third most troublesome aspect of depression, behind low libido and weight gain but ahead of sad mood, low energy, poor sleep, and many other symptoms that might be considered more characteristic of depression. The same study found reason to believe patient reports: patients’ self-reported assessment of memory problems was correlated with performance on recollection-based memory tests, which depend heavily on hippocampal function. In other words, the participants thought that their memories were failing, and they were right (but see Mowla et al., 2008, for evidence that depressed adults have limited insight into the extent of their memory deficits).

A new field focused on memory therapeutics for depression is emerging in response to findings like these (for review, see Dalgleish and Werner-Seidler, 2014; for a review of work directed at improving working memory in depressed adults, see (Becker et al., 2015). The field is oriented around the fact that autobiographical memory retrieval has several striking qualities in depressed adults. First, it is oriented toward emotionally negative material. Second, it is frequent overly general: given a cue and the explicit instruction to retrieve a specific, time-limited memory, depressed adults are instead prone to recall categorical memories that span several discrete events and that often have a negative theme. Third, autobiographical memory retrieval is apt to set off a downward spiral of rumination and self-recrimination, as the depressed individual perseverates on past failures and fails to see how events may play out differently in the future. The field of memory therapeutics takes these qualities of autobiographical memory retrieval as targets, training depressed adults to rapidly retrieve positive memories and elaborate upon them, being as specific as possible and avoiding cycles of negative rumination. Furthermore, when unintentional retrieval of negative memories does occur, clients are instructed to regard them with the non-judgmental, accepting perspective taught in mindfulness-based cognitive therapy (Teasdale et al., 2000), as a way to defuse the memories’ emotional charge. Although the field of memory therapeutics is very new, there is already some evidence that these methods are clinically effective (e.g., Watkins et al., 2009, 2012; Neshat-Doost et al., 2012).

The proposal developed here is complementary to work on memory therapeutics because its focus is different. Memory therapeutics are primarily aimed at improving the precision and selectivity of retrieval, whereas the hypothesis advanced here proposes that dopamine dysfunction compromises memory formation. If this hypothesis proves true, there is no reason why a clinician could not target both encoding and retrieval for maximum benefit. One can imagine a scenario in which improved dopaminergic tone in mesolimbic circuits could enhance reward responses in depressed adults, boosting late-LTP and thus improving long-term memory for positive events. Simultaneously, a memory therapeutics approach focused on directing retrieval searches toward concrete, specific, positive material from a client’s life could enhance positive mood while simultaneously decreasing the propensity to ruminate on overgeneral negative memories. Together, these two strategies could have a powerful effect on mood that would place clients on an upward spiral toward better outcomes.

## Qualifications and a Role for Dopamine in Negative Memories

In order to advance the central argument of this article, I have glossed over several important points that deserve mention. First, this proposal should not be read as equating depression with dopamine dysfunction. Depression is a heterogeneous condition and a diagnosis of MDD can reflect a wide range of symptoms, many of which have little (if anything) to do with dopamine. Thus, this proposal should be narrowly read: it is strictly about how dopamine dysfunction may compromise memory persistence in depression. Second, the mechanism proposed here is complementary to the hippocampal stress hypothesis, because stress is thought to cause dopaminergic abnormalities that are central to depression (Dillon et al., 2014b). In other words, hippocampal volume reductions and reward-based memory deficits may both be downstream consequences of stress, and it may be useful to determine whether and how they interact with one another (i.e., do changes in D1/D5 receptor distributions figure in hippocampal volume reductions?), particularly because recent rodent studies indicate that antagonizing glucocorticoid receptors can disrupt the acquisition, retrieval, and reconsolidation of conditioned place preferences for rewarding events (Dong et al., 2006; Fan et al., 2013; Achterberg et al., 2014). Third, chronic stress models used to induce anhedonia in rodents result in tonically reduced dopamine concentrations (Willner, 2005), but memory for individual events is probably influenced by phasic dopamine bursting. How tonic and phasic dopamine levels interact to influence memory retention is unclear and an important topic for future study (Shohamy and Adcock, 2010). Fourth, this proposal is focused on encoding and consolidation, but it will be important to examine memory retrieval in depression as well, as the discussion of memory therapeutics implies. Healthy adults retrieve positive memories to repair negative moods, and in doing so they activate the striatum and the medial PFC (Speer et al., 2014), two regions that figure prominently in mechanistic accounts of depression (Pizzagalli et al., 2009; Lemogne et al., 2012). Thus, poor memory for positive material in depression may frequently reflect problems with retrieval. Indeed, as retrieval depends heavily on PFC function (Dobbins et al., 2002) and depression is characterized by hypofrontality (e.g., Mayberg et al., 1994; Pizzagalli, 2011), problems mounting successful retrieval attempts may be a general issue in depressed adults, extending beyond memory for positive material. Fifth, although there is a wealth of evidence indicating that depression is typically associated with weak responses to rewarding stimuli and dysfunction in brain reward systems (Treadway and Zald, 2011; Pizzagalli, 2014; Whitton et al., 2015), evidence linking these findings to dopamine is usually indirect and often based on inference from studies in non-human animals, thus the link between anhedonic symptoms of depression and dopamine needs strengthening. Other neurochemical systems, including the opioid, endocannabinoid, serotonergic, and glutamatergic systems, are likely relevant, and of course humans experience a diverse range of positive emotions that may be more (e.g., excitement) or less (e.g., tranquility) tightly linked to phasic dopamine bursting. Similarly, the account of LTP offered earlier amounts to a thumbnail sketch, as the process of memory formation is remarkably complex and involves many factors at the molecular level. Nonetheless, dopamine appears to play a central role in anhedonic depression and the transition from early-to-late LTP, and thus it is an excellent starting point for a mechanistic account of positive memory deficits in depression.

Finally, it is very clear in the behavioral neuroscience literature that the positive effects of dopamine on retention are not limited to memory for positive events (e.g., Zweifel et al., 2011), although it may be most easily detected and studied in such cases. As an example, Sariñana et al. (2014) showed that D1 receptors in the dentate gyrus are crucial for contextual fear conditioning in mice. They created D1 and D5 receptor knockouts and administered shock in one box (context A). 24 h later, the mice were exposed to the original box and another, similar box (context B). D5 knockouts and control mice froze readily in context A but not context B, consistent with contextual fear conditioning, but D1 knockouts did not discriminate—they froze to a similar degree in both contexts. Similarly, markers of early gene activity (c-fos counts) in the dentate gyrus told a similar tale: D5 knockouts discriminated between their home cage, context A (where they had been shocked), and an entirely novel context, but no such differentiation was seen in the D1 knockouts. Consistent with many findings reviewed earlier, no deficit was seen in D1 knockouts when memory was tested one to 3 h after acquisition, implying that the effect is specific to memory persistence, and no deficit was seen in amygdala-dependent cue-based fear conditioning, implying that the effect is dependent on D1 receptors in the hippocampus. In short, this study provided evidence that D1 receptors in the dentate gyrus are critical for contextual fear conditioning.

In the current context, I wish to use the technically elegant work of Sariñana et al. (2014) to make a simpler point: despite the strong link between dopamine and reward, this study underscores the fact that dopamine (and D1 receptors in particular) can be important for retaining memory for aversive experiences so long as they depend on hippocampal activation. Presumably these findings depend on the subset of midbrain dopaminergic neurons that respond to salient stimuli whether those stimuli are rewarding or punishing (Matsumoto and Hikosaka, 2009; Bromberg-Martin et al., 2011). Consequently, although studying interactions between dopamine networks and the MTL memory system may prove especially valuable for understanding positive memory deficits in depression, it may help explain poor memory more broadly. Along these lines, an intriguing study in rodents found that if phasic dopamine bursting is blocked, a simple light-shock fear conditioning paradigm can result in behavior consistent with generalized anxiety, presumably because the tight relationship between illumination of the light and shock delivery is not well-encoded, leading to overgeneralization of the fear response (Zweifel et al., 2011).

## Conclusion

Depressed adults typically present with episodic memory deficits, and they rate these deficits as a particularly troublesome aspect of the illness (MacQueen et al., 2002). Furthermore, memory for positive material is especially impaired in depression but the neural mechanisms responsible for this deficit are not well characterized. I propose that poor memory for positive material in depression emerges because of anhedonia and its association with dysfunction in mesolimbic dopamine networks widely associated with reward processing.

Although there is little direct work on this topic, there is a compelling body of evidence, across several levels of analysis, implicating dopamine transmission in memory persistence. The STC hypothesis presents dopamine as the instigator of protein synthesis that cements the transition from early- to late-LTP in hippocampal neurons. Experiments probing episodic memory in rodents show that blocking D1/D5 receptors in the hippocampus during encoding has little effect on tests of immediate memory but exerts a powerfully negative effect on delayed tests. By contrast, administration of D1/D5 agonists and exposure to novelty reliably boost memory retention. A growing literature in healthy humans is consistent with findings in rats and hippocampal slices, as reward anticipation and novelty exposure enhance encoding and retention. These mechanisms are space-saving devices: the brain can only store so much material, and it gives privileged access to events that are proximal to dopamine release. In this way, episodes that culminate in reward delivery are well-retained, presumably to allow the organism to behave adaptively should similar circumstances arise in the future.

In sum, there is a widespread consensus that anhedonic depression is associated with dysfunction in brain reward circuitry, with an emphasis on stress-induced disruption of the mesolimbic dopamine system. Initial results suggest that this has consequences for memory, as one would predict based on the molecular, behavioral, and human neuroscience literatures. Given the potential to make a meaningful difference in the way memory problems in depression are understood and treated, and in light of the considerable supporting evidence marshaled here, thoroughly testing and refining this proposal would constitute time and effort well-spent.

### Conflict of Interest Statement

The author declares that the research was conducted in the absence of any commercial or financial relationships that could be construed as a potential conflict of interest.

## Footnotes

1 For brevity, I will use the terms “depression” and “memory” to refer to unipolar depression and episodic memory, respectively, throughout the remainder of the manuscript unless otherwise noted.
